# Vancomycin-Loaded Collagen/Hydroxyapatite Layers Electrospun on 3D Printed Titanium Implants Prevent Bone Destruction Associated with *S. epidermidis* Infection and Enhance Osseointegration

**DOI:** 10.3390/biomedicines9050531

**Published:** 2021-05-10

**Authors:** Tomáš Suchý, Lucie Vištejnová, Monika Šupová, Pavel Klein, Martin Bartoš, Yaroslav Kolinko, Tereza Blassová, Zbyněk Tonar, Marek Pokorný, Zbyněk Sucharda, Margit Žaloudková, František Denk, Rastislav Ballay, Štefan Juhás, Jana Juhásová, Eva Klapková, Lukáš Horný, Radek Sedláček, Tomáš Grus, Zdeněk Čejka, Zdeněk Čejka, Kateřina Chudějová, Jaroslav Hrabák

**Affiliations:** 1 Department of Composites and Carbon Materials, Institute of Rock Structure and Mechanics, Czech Academy of Sciences, 18209 Prague 8, Czech Republic; supova@irsm.cas.cz (M.Š.); sucharda@irsm.cas.cz (Z.S.); zaloudkova@irsm.cas.cz (M.Ž.); denk@irsm.cas.cz (F.D.); 2 Faculty of Mechanical Engineering, Czech Technical University in Prague, 16000 Prague 6, Czech Republic; Lukas.Horny@fs.cvut.cz (L.H.); Radek.Sedlacek@fs.cvut.cz (R.S.); 3 Biomedical Center, Faculty of Medicine in Pilsen, Charles University, 30100 Pilsen, Czech Republic; Lucie.Vistejnova@lfp.cuni.cz (L.V.); Pavel.Klein@lfp.cuni.cz (P.K.); Martin.Bartos@lf1.cuni.cz (M.B.); Yaroslav.Kolinko@lfp.cuni.cz (Y.K.); Tereza.Blassova@lfp.cuni.cz (T.B.); Zbynek.Tonar@lfp.cuni.cz (Z.T.); CHUDEJOVAK@fnplzen.cz (K.C.); jaroslav.hrabak@lfp.cuni.cz (J.H.); 4 Department of Histology and Embryology, Faculty of Medicine in Pilsen, Charles University, 301 00 Pilsen, Czech Republic; 5 Institute of Dental Medicine, First Faculty of Medicine, Charles University and General University Hospital in Prague, 12000 Prague 2, Czech Republic; 6 Institute of Anatomy, First Faculty of Medicine, Charles University, 12000 Prague 2, Czech Republic; 7 R&D Department, Contipro Inc., 56102 Dolni Dobrouc, Czech Republic; Marek.Pokorny@contipro.com; 8 1st Department of Orthopedics, First Faculty of Medicine, Charles University in Prague and Motol University Hospital, 150 06 Prague 5, Czech Republic; ballayrastislav@gmail.com; 9 PIGMOD Centre, Laboratory of Cell Regeneration and Plasticity, Institute of Animal Physiology and Genetics, Czech Academy of Sciences, 27721 Libechov, Czech Republic; Juhas@iapg.cas.cz (Š.J.); juhasova@iapg.cas.cz (J.J.); 10 Department of Medical Chemistry and Clinical Biochemistry, Charles University, 2nd Medical School and University Hospital Motol, 15006 Prague 5, Czech Republic; eva.klapkova@email.cz; 11 2nd Department of Cardiovascular Surgery, First Faculty of Medicine, Charles University and General University Hospital in Prague, 12000 Prague 2, Czech Republic; Tomas.Grus@vfn.cz; 12 ProSpon Ltd., 27201 Kladno, Czech Republic; zdenek.cejka_ml@prospon.cz (Z.Č.J.); zdenek.cejka@prospon.cz (Z.Č.)

**Keywords:** orthopedic implant, collagen, hydroxyapatite, vancomycin, implant-related bone infection, *Staphylococcus epidermidis*, osseointegration, bone, rat, minipig

## Abstract

The aim of the study was to develop an orthopedic implant coating in the form of vancomycin-loaded collagen/hydroxyapatite layers (COLHA+V) that combine the ability to prevent bone infection with the ability to promote enhanced osseointegration. The ability to prevent bone infection was investigated employing a rat model that simulated the clinically relevant implant-related introduction of bacterial contamination to the bone during a surgical procedure using a clinical isolate of *Staphylococcus epidermidis*. The ability to enhance osseointegration was investigated employing a model of a minipig with terminated growth. Six weeks following implantation, the infected rat femurs treated with the implants without vancomycin (COLHA+*S. epidermidis*) exhibited the obvious destruction of cortical bone as evinced via a cortical bone porosity of up to 20% greater than that of the infected rat femurs treated with the implants containing vancomycin (COLHA+V+*S. epidermidis*) (3%) and the non-infected rat femurs (COLHA+V) (2%). The alteration of the bone structure of the infected COLHA+*S. epidermidis* group was further demonstrated by a 3% decrease in the average Ca/P molar ratio of the bone mineral. Finally, the determination of the concentration of vancomycin released into the blood stream indicated a negligible systemic load. Six months following implantation in the pigs, the quantified ratio of new bone indicated an improvement in osseointegration, with a two-fold bone ingrowth on the COLHA (47%) and COLHA+V (52%) compared to the control implants without a COLHA layer (27%). Therefore, it can be concluded that COLHA+V layers are able to significantly prevent the destruction of bone structure related to bacterial infection with a minimal systemic load and, simultaneously, enhance the rate of osseointegration.

## 1. Introduction

The increase in the number of primary endoprosthesis implantations has led to a corresponding increase in the occurrence of post-operative complications. Infection features high on the list of the most common reasons for the necessity for revision surgery along with osteolysis and aseptic loosening and patient- and implant-related problems [1]. Direct contamination, contamination via the blood stream or from a site contiguous to the implant may lead to the development of the infection of the bone tissue (e.g., osteomyelitis) [2]. Depending on the rate of infection development, it can be treated via routine antimicrobial therapy or the radical debridement of the necrotic and infected tissue accompanied by extensive doses of antibiotics [2]. To date, no method has been generally accepted as the method of first choice with concern to the treatment of osteomyelitis [3]. The localized delivery of antibiotics overcomes the problem associated with the limited accessibility of systemically administered drugs to the infected site [4]. Antibiotics can be tethered directly to a titanium alloy surface. The modification of titanium surfaces with antibiotics has focused, to date, predominantly on vancomycin [5,6,7,8,9,10]. However, the main drawback of covalent bonding is the requirement for the treatment of the alloy surface with methacryloxypropyltrimethoxysilane [8] or bis(ethylene glycol) linkers [10] so as to enable the subsequent attachment of antibiotics (ATB). In contrast to the low level of efficiency of poly (methyl methacrylate) (PMMA) antibiotic carriers, which have been used in the field of orthopedics for almost 40 years [2,11], biodegradable and resorbable materials enjoy the advantages of the controlled release of antibiotics, broad compatibility with a range of antimicrobial agents, and the dispensing of the need for follow-up surgery for the removal of non-biodegradable carriers [12,13,14,15,16,17]. The application of biomaterials in the treatment of implant-associated osteomyelitis was recently comprehensively reviewed by Inzana et al. [2]. Ceramic materials such as calcium sulphate [18], calcium phosphates [19,20], synthetic polymers such as poly(d,l-lactide) [21], poly(d,l-lactide-co-glycolide) [22,23], or natural polymers such as collagen [24], chitosan [25], fibrin [26], and their combinations in the form of composite materials [3,27,28,29,30] all constitute clinically used or experimentally developed biodegradable local carriers of antibiotics. However, only a limited number of complex in vivo studies have been conducted to date on a combination of collagen/calcium phosphate/vancomycin [3,27,30,31,32,33,34,35,36].

Of those studies, a number have already evaluated systems that employ such materials by means of cellular in vitro testing. Pon-On et al. [32] studied a drug delivery vehicle consisting of spherical calcium phosphate-collagen particles covered with flower-like blossoms loaded with vancomycin. Their study determined that the loading efficiency of this delivery system was around 78% in cases where immersion in three various media lasted over 2 weeks. The cellular response was evaluated employing rat osteoblast-like UMR-106 cells. Coelho et al. [35] developed an innovative controlled release system based on heparinized nanohydroxyapatite/collagen that allowed for the more sustainable and controlled release of vancomycin (360 h, i.e., 15 days) than that of non-heparinized granules. However, after 216 h, the concentrations were found to be lower than the minimum inhibition concentration (MIC). They also proved that the presence of vancomycin did not affect the viability of MC3T3-E1 cells after 14 days of culturing. Other studies applied a more comprehensive evaluation of biocompatibility using animal in vivo tests. Lian et al. [31] synthetized an antibacterial bone graft based on nano-hydroxyapatite/collagen/poly(lactic acid) loaded with vancomycin. They studied the delivery of vancomycin from a composite for up to 4 weeks. Biocompatibility in vitro was evaluated using rabbit marrow stromal cells. In vivo biocompatibility was evaluated by means of implantations in Japanese white rabbits for one and three months, albeit only subcutaneously. The same research group [37] also developed a nano-hydroxyapatite/collagen vancomycin carrier supplemented with α-calcium sulphate hemihydrate. The inhibition ratio (*S. aureus*) of this composite was found to be more than 99.8% for 16 h of incubation. After 17 days of incubation at 37 °C in vitro, the concentration of vancomycin in the elution fluid was around 12 μg/mL. A subsequent study by the same group [34] investigated the adhesion and proliferation of murine osteoblastic MC3T3-E1 cells on the same material system. The degree of effectiveness with respect to restoring infectious bone defects was evaluated in vivo using a New Zealand rabbit model of chronic osteomyelitis caused by MRSA. After 12 weeks of implantation, it was proved that the infected bone tissues without drug release had been destroyed in various parts of the rabbit femurs, while with concern to the treatment group the extent of bone reconstruction was better than that of the control group and, moreover, closely resembled normal bone.

Since the ideal approach to the treatment of infected bone defects consists of the simultaneous repair of large-size bone defects and the inhibition of related infections, the use of an osteoconductive bone graft with antibiotic and growth factor release capabilities and osteogenesis-matched degradation properties is possible [38]. This study comprises a follow-up to previous papers published by the research team [3,27,30,39] that addressed the development of a coating for orthopedic implants in the form of a nanofibrous layer, which exerts a strong local anti-infection effect and, simultaneously, does not lead to a decrease in the rate of osseointegration necessary for the suitable fixation of the implant. A comprehensive evaluation was conducted of the vancomycin release kinetics, antimicrobial efficiency, and cytocompatibility of such collagen/hydroxyapatite/vancomycin layers. The resulting nanostructured layers prepared by means of electrospinning from a collagen/hydroxyapatite dispersion, subsequently cross-linked with EDC/NHS and finally impregnated with vancomycin provided for effective antibiotic release kinetics above the MIC for VRSA in human blood plasma for up to 30 days and exhibited both an inhibitory effect against populations of *S. aureus*, *S. epidermidis*, and *E. faecalis* and a sufficient degree of cytocompatibility with no indication of cytotoxic effects when using human osteoblastic cells in direct contact with the layers or in 24-h infusions thereof [27].

The aim of the study was to perform a comprehensive in vivo biological assessment of vancomycin-loaded collagen/hydroxyapatite layers (COLHA+V) that were electrospun directly on titanium implants. Firstly, the ability of COLHA+V layers to prevent bone infection accompanied by bone structure destruction was investigated employing a rat model that simulated the clinically relevant implant-related introduction of bacterial contamination to the bone during a surgical procedure using a clinical isolate of *S. epidermidis* that originated from a rejected human patient implant. Secondly, the ability of the COLHA layers to enhance osseointegration was investigated employing a large animal model of a minipig with terminated skeleton growth. To the best of our knowledge, no such comprehensive in vivo evaluation has been employed to date with concern to a collagen/calcium phosphate/vancomycin material combination.

## 2. Materials and Methods

### 2.1. Preparation of the Collagen/Hydroxyapatite Electrospun Layers

The collagen/hydroxyapatite layers (COLHA) were prepared via the electrospinning of an 8 wt % collagen (type I, source: calf skin, VUP Medical, Brno, Czech Republic) PBS/ethanol solution (1/1 *v*/*v*) modified with 8 wt % (to the collagen) of polyethylene oxide (PEO; Mr 900,000, Sigma Aldrich, St. Louis, MI, USA) with dispersed 15 wt % of hydroxyapatite (HA) particles (85/15; collagen/HA). The HA nanoparticles (average 150 nm, Sigma Aldrich, Germany) were used as received. Electrospun mats were prepared using a high voltage level of 45 kV and the feeding rate was set at 130 μL·min^−1^, the temperature at 24 °C and the relative humidity at 20–25% (4SPIN, Contipro, Dolni Dobrouc, Czech Republic). The production rate of the COLHA mats was increased via the application of electroblowing [40]; the flow rate of the preheated air (25 °C) was set at 30 L·min^−1^. The stability of the COLHA layers deposited on the implants (see Section 2.2) was enhanced by means of cross-linking with a 95% ethanol solution containing N-(3-dimethylaminopropyl)-N-ethylcarbodiimide hydrochloride (EDC) and N-hydroxysuccinimide (NHS) at a weight ratio of 4:1; the EDC and NHS (Sigma Aldrich, Germany) were used as received. Following a reaction period of 24 h at 37 °C, all the layers were washed in 0.1 M Na_2_HPO_4_ (2 × 45 min) and rinsed using deionized water (30 min); the PEO and NaCl were fully leached out [27]. They were then frozen at −15 °C for 5 h and lyophilized (BenchTop 4KZL, VirTis, Los Angeles, CA, USA). The cross-linked layers were further impregnated (COLHA+V) with a vancomycin (vancomycin hydrochloride, Mylan S.A.S, St Priest, France) ethanol solution and dried at room temperature in a laminar box until a constant weight was attained (up to 2 h). The final concentration of vancomycin in the COLHA layers consisted of 10% of the weight thereof.

### 2.2. Preparation of the Titanium Implants

**Figure 1:**
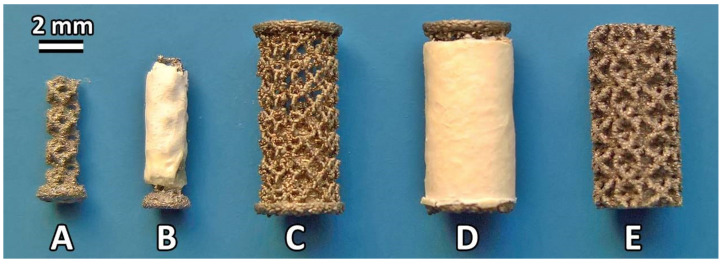
Representative images of the titanium implants. Ti printed implant before (**A**) and after (**B**) the deposition of a COLHA+V layer intended for use in the antimicrobial activity experiment; Ti printed implant before (**C**) and after (**D**) the deposition of a COLHA+V layer intended for use in the osseointegration experiment; Ti printed control sample (**E**).

COLHA and COLHA+V layers were deposited upon two different printed titanium implants intended for implantation in two subsequent in vivo experiments (in vivo infection prevention and osseointegration). Three-dimensional printed samples were prepared from standard implantable concept laser titanium alloy—CL 41 ELI (Ti6Al4V) powder (particle size 15–63 μm) with a trabecular structure designed using Magics computer-assisted design (CAD) software (Materialise, Leuven, Belgium). The implants were fabricated by means of an SLM system (M2 Cusing Laser 1x200 W, Concept Laser GmbH, Lichtenfels, Germany) based on the CAD (Magics, version 19.02, Leuven, Belgium) data with a trabecular Dode-thick (MSG) shape and each with trabecula dimensions of 1.5 mm × 1.5 mm × 1.5 mm. The implants intended for the antimicrobial experiment in rats were of a nail-like shape (see Figure 1A,B) with an outer diameter of 2 mm, an inner diameter of 1.5 mm and a length of 5.5 mm.

The nail-like shape ensured the protection of the electrospun layers when inserting the implant into the bone cavity. The implants intended for the osseointegration experiment in pigs were of a spool-like shape for the same reason. These samples had an outer diameter of 4 mm, an inner diameter of 3.5 mm, and a length of 8 mm (see Figure 1C). The deposited COLHA and COLHA+V electrospun layers finally filled the inner diameter of the implants up to 4 mm (Figure 1C). The Ti printed samples (CP) were prepared with the same structure and with a diameter of 4 mm and a length of 8 mm (Figure 1E). All the titanium alloy samples intended for the osseointegration experiment (Figure 1C–E) were prepared with an M2.2 inner metric thread for the fixation of the insertion jig used in the implantation procedure. All the samples were ultrasonically washed with ethanol and distilled water and dried at 60 °C overnight prior to the deposition of the electrospun layers. Following deposition and impregnation, all the samples were packed and sealed in indicator bags and exposed to sterilization at a nominal dose of 25 kGy (BIOSTER, a.s., Veverská Bítýška, Czech Republic).

### 2.3. Characterization of the Selected Strain of Staphylococcus epidermidis

A *Staphylococcus epidermidis* isolate was recovered from the blood culture of a patient suffering from a catheter-related infection. The identification of the isolate was performed by means of MALDI-TOF mass spectrometry using a MALDI Biotyper^®^ module (Bruker Daltonics, MA, USA). Susceptibility to 12 antibiotics was determined via the microdilution broth method according to EUCAST guidelines [41] and interpreted using EUCAST standards version 10.0 [42]. The ability to form a biofilm was detected via the Christensen method as modified by Ruzicka et al. [43].

### 2.4. The Experimental Rat Model Simulating the Clinically Relevant Introduction of Bacterial Infection to the Bone

In order to replicate a case of bone infection following primary implantation, we applied a clinical isolate of *S. epidermidis* from a rejected implant for the induction of infection in the femur of a rat model. The experiment was approved by the Animal Welfare Advisory Committee of the Ministry of Education, Youth and Sports of the Czech Republic (approval ID MSMT-249/2017-2). Thirty male Wistar rats aged 5 months (Masaryk University Brno, Brno, Czech Republic) were divided into 3 groups, i.e., a group with implanted COLHA+V layers without infection (*n* = 10), a group with implanted COLHA layers with the *S. epidermidis* infection (*n* = 10), and a group with implanted COLHA+V layers with the *S. epidermidis* infection (*n* = 10). The experimental surgical procedure was performed under general anesthesia. The animals were subjected to light inhalation anesthesia induced by isoflurane in oxygen using an anesthetic machine (Vetnar 1100, Grimed, Lhota pod Radcem, Czech Republic) connected to an inhalation chamber. Following the elevation of the abdominal wall of the restrained animal, the anesthetic mixture was carefully injected into the peritoneal cavity so as not to affect the intestine. The anesthetic mixture was prepared prior to each experiment in a syringe via the mixing of propofol (100 mg/kg; Propofol 2%, Fresenius Kabi, Bad Homburg vor der Höhe, Germany), medetomidine (0.1 mg/kg; NarcoStart^®^, Produlab Pharma B.V., Raamsdonksveer, Netherlands), and nalbuphine (0.1 mg/kg; Nalbuphin Orpha, Orpha-Devel Handels und Vertriebs GmbH, Purkersdorf, Austria). The anesthetized animals were placed on a tempered operating table and continually supplied with oxygen via a face mask and monitored by means of pulse oximetry. During the operational procedure, the COLHA and COLHA+V layers were impregnated with 2 μL of a clinical isolate *S. epidermidis* suspension (1.0 McFarland) so as to simulate a clinical situation in which an infection is accidentally introduced into the surgical field. One of the hind legs was shaved and disinfected, the animal was placed in the dorsal position and the pelvis positioned so as to provide comfortable lateral access to the femur. Following cutaneous incision, access was gently prepared between the hind leg vastus fibularis and biceps femoris muscles so as to expose the femur. A hole (2 mm × 5 mm) was then drilled into the femur and completed using a surgical milling cutter and Kirschner wire in order to enable the complete insertion of the implants into the medullary cavity. One of the implants was loosely inserted into a slightly larger prepared hole in the medullar cavity of the left femur of each animal. The hole was then sealed with PALACOS^®^ (Heraeus Medical GmbH, Wehrheim, Germany) bone cement. The muscles, fascia, and skin were treated separately with an absorbable suture and the surgical wound covered with Novikov solution. The general anesthesia was then terminated by means of an IM injection of atipamezole (0.5 mg/kg; NarcoStop^®^, Produlab Pharma B.V., Raamsdomksveer, The Netherlands). Following surgery, the animals were provided with analgesia for 4 days using tramadol (12.5 mg/kg; Tramal^®^, STADA Arzneimittel AG, Bad Vilbel, Germany) and carprofen (5 mg/kg; Rimadyl^®^, Zoetis, Sao Paulo, Brazil) as recommended by Cannon et al. [44]. Subsequently, blood samples were collected from the tail vein at weekly intervals. Blood collection for the determination of the vancomycin concentration released from the implants was performed under light isoflurane anesthesia (3% isoflurane in O_2_) administered via a face mask. The animals were sacrificed after 6 weeks and femur samples were collected for histological and micro-CT analysis purposes. The rats were kept under conventional conditions (12/12 dark/light cycle) during the experiment according to EU directive 2010/63/EU in sterile polycarbonate microisolators (Bioscape, Castrop-Rauxel, Germany) with bedding (Lignocel Select fine, JRS, Rosenberg, Germany) and free access to water and pelletized feed (ST1, Velaz, Praha-Lysolaje, Czech Republic).

### 2.5. Bone Histology of the Rat Model

The samples were processed into ground sections as described previously [45,46] for the purpose of the histological analysis. Briefly, the samples were fixed with a 10% formaldehyde solution (7 days), immersed in 70% ethanol and then micro-CT scanned (see the next paragraph). The samples were further dehydrated in ascending grades of ethanol and embedded for two days in methyl methacrylate (Merck Millipore, Darmstadt, Germany) without a polymerization initiator. The samples were then emplaced in molds with resin and polymerized. The polymerization of the resin was initiated using benzoyl peroxide (Merck Millipore, Darmstadt, Germany). The blocks with the samples were then sectioned perpendicularly along the long axis of the implants. The samples were cut using a diamond disc, the cutting area of the blocks was ground using a sequence of abrasive papers, and, finally, the samples were polished. A clean slide was then glued to the polished side and a second section was made approximately 300–500 μm from the slide and parallel to it. The slide with the section was ground once more to a final thickness of between 70 and 90 μm. The final polishing of the surface involved the use of paper coated with a fine textile cloth and 3 μm diamond paste. The papers used for grinding and polishing were positioned on a rotation desk with the samples pressed towards the desk (EcoMet™ 250, Struers, Ballerup, Hovedstaden, Denmark). Finally, the sections were stained with 20% Giemsa’s azur eosin methylene blue solution (Merck Millipore, Darmstadt, Germany). The whole surface of the implant and the surrounding area was photographed using a bright field microscope with a 10× objective. We compared changes in the surrounding bone structure and the grade of infiltration by inflammatory cells by actively searching for signs of foreign-body cell reactions, insufficient osseointegration, osteolysis, periprosthetic infection, and necrosis.

### 2.6. Micro-CT Analysis of Rat Model

Micro-CT scans were acquired ex-vivo using a SkyScan 1272 (Bruker micro-CT, Kontich, Belgium) micro-CT device. Each of the femurs with inserted implants were mounted on a micro-stage and scanned immersed in 70% ethanol in a plastic tube. The long axes of the samples were oriented vertically. The scanning parameters were as follows: 10 µm pixel size, rotation step = 0.2°, camera binning 2 × 2, source voltage 100 kV, source current 100 µA, 0.11 mm Cu filter, frame averaging = 2, and 360° rotation; the scanning time was approximately 3 h and 30 min for each specimen. The flat-field correction was updated prior to each acquisition. Cross-section images were reconstructed from the projection images using NRecon software (Bruker micro-CT, Kontich, Belgium) with a modified Feldkamp algorithm. Correction procedures were applied (misalignment, ring artefact, and beam hardening correction) so as to reduce the effect of computed tomography artefacts. Visualizations were acquired by means of DataViewer (2D cross-section images) and CTVox (3D images; Bruker, Kontich, Belgium). The 3D analysis of the structure of the rat femurs with the implants (*n* = 10) was conducted by means of CTAn (Bruker, Kontich, Belgium). In order to evaluate the changes in the bone structure, a region of interest was created in each specimen in the area surrounding the implant. The regions of interest were hand drawn in cross-sectional images and comprised cortical bone with the exclusion of the implant and cement material. The vertical dimension of the volume of interest (VOI) was defined via the length of the implant. The VOI was subjected to 3D analysis following the application of an image noise reduction procedure (based on image filtering in 3D) and binarization. Image processing prior to the analysis was optimized using TeIGen software (test image generator [47], https://mjirik.github.io/teigen, accessed on 24 January 2019). The results were presented in the form of cortical bone porosity (1):

(1) $$P\;\left(\%\right)=\frac{\mathrm{Total}\;\mathrm{porosity}\;\mathrm{volume}}{\mathrm{VOI}\;\mathrm{volume}}$$

### 2.7. SEM and Elemental Analysis of Rat Model

The chemical composition of the bone mineral in the explanted rat femurs was verified by means of energy dispersive X-ray spectroscopy (EDS) using an EDS SDD EDAX Apollo (EDAX Genesis system, Mahwah, NJ, USA) detector on a scanning electron microscope (SEM; Quanta 450 Microscope, FEI, Hillsboro, OR, USA) in the high vacuum mode. The histological thin sections were carbon-coated on a K550X (Quorum Technologies, Kent, UK) sputter coater in an argon atmosphere prior to the analysis. The concentrations of elemental Ca and P and the Ca/P molar ratio were determined aimed at the assessment of changes in the bone mineral Ca/P weight ratio that reflected inflammatory processes. Kourkoumelis et al. [48] have used the EDS method for the evaluation of the Ca/P ratio at different sites in normal and osteoporotic rabbit bones. They proved that EDS provides a suitable analytical method for the in vitro quantification of the Ca/P ratio, that it demonstrates a high enough degree of precision for the taking of semiquantitative measurements and that it allows for a better statistical significance for the Ca/P ratio than does error-prone simple composition assessment. Ten measurements were carried out at different locations on the surface of the bone for each of the eight histological thin sections and for each group of implants.

### 2.8. Vancomycin Concentration in Blood Plasma of Rat Model

Blood samples were taken 7 and 14 days following surgery from the tail vein under light isoflurane anesthesia (3% isoflurane in O_2_) administered via a face mask. The blood samples were centrifuged (3000× *g*, 10 min) and the plasma samples stored at −80 °C. Prior to the analysis, the samples were thawed and the vancomycin was quantified by means of high performance liquid chromatography (HPLC). The solid phase extraction method and HPLC analysis (HPLC on an Agilent 1260 series system equipped with a DAD diode array detector, Agilent Technologies, Santa Clara, CA, USA) were employed for the characterization of the in vivo release rates of the vancomycin and its crystalline degradation antibiotically inactive products [48] from the implanted samples to the bloodstream in order to determine a possible systemic load. The HPLC method is described in an article written by Melichercik et al. [49].

### 2.9. The Minipig Model for Analysis of Osseointegration of COLHA Layers

All the experiments were carried out according to guidelines for the care and use of experimental animals as approved by the Resort Professional Commission of the CAS for the Approval of Projects with Experiments on Animals (approved protocol no. 07/2017). The study involved the use of three minipigs (females) aged 30–48 months and with body weights of 90 ± 20 kg. The general anesthesia of the miniature pigs was induced via the intramuscular injection of a TKX mixture (tiletamine 2 mg/kg + zolazepam 2 mg/kg + ketamine 2 mg/kg + xylazine 0.4 mg/kg). Six cylindrical samples of 4 mm in diameter and 8 mm in length were implanted under the proximal condyle of the right and left femurs under sterile conditions and guided by the general anesthesia of the animals (isoflurane, N_2_O, propofol, fentanyl, and pancuronium bromide). Six control (CP) and six experimental (COLHA, COLHA+V) samples were implanted in each of the three animals for six months. All the implants were press-fit inserted (i.e., without a gap between the bone and the implant). The operational procedure was as follows: firstly, a longitudinal skin cut was made followed by a cut across the fascia in the proximal condyle area. The intramuscular septum was opened by means of the introduction of Hohmann bone elevators. The periosteum was removed using a Sauerbruch raspatory. Six holes with dimensions of 3.8 mm in diameter and a length of 10–12 mm were drilled using a surgical drill with continuous cooling via the application of a physiological solution. Once the holes had been prepared and rinsed with the physiological solution, the implants were inserted. The operation space was then cleaned using the physiological solution prior to hypodermis and skin suture, immediately following which X-ray images of the implant sites were taken using a C-arm ARCADIS Varic (Siemens, München, Germany). The animals were treated with ATB (Eficur and Betamox, Norbrook, Newry, Northern Ireland) and a non-steroidal anti-inflammatory drug (Flunixin, Norbrook, Newry, Northern Ireland) during the perioperative period and for the first four days after implantation. The animals were bred under standard conditions according to the rules applicable for the breeding of laboratory animals in the Czech Republic. Six months following implantation, the pigs were sacrificed via exsanguination under deep general anesthesia conditions and the implants removed and fixed in a 10% formalin solution.

### 2.10. Bone Histology of Minipig Model

The samples were processed using the same procedure as that applied to the rat femurs (see Section 2.5). Sections of the pig femurs were stained using 20% Giemsa’s azur eosin methylene blue solution (Merck Millipore, Darmstadt, Germany) [50] and the whole surface of the implants photographed using a bright field microscope with a 10× objective. All the steps applied during the microscopic evaluation complied with ISO 10993-6.

### 2.11. Micro-CT Analysis of Minipig Model

**Figure 2:**
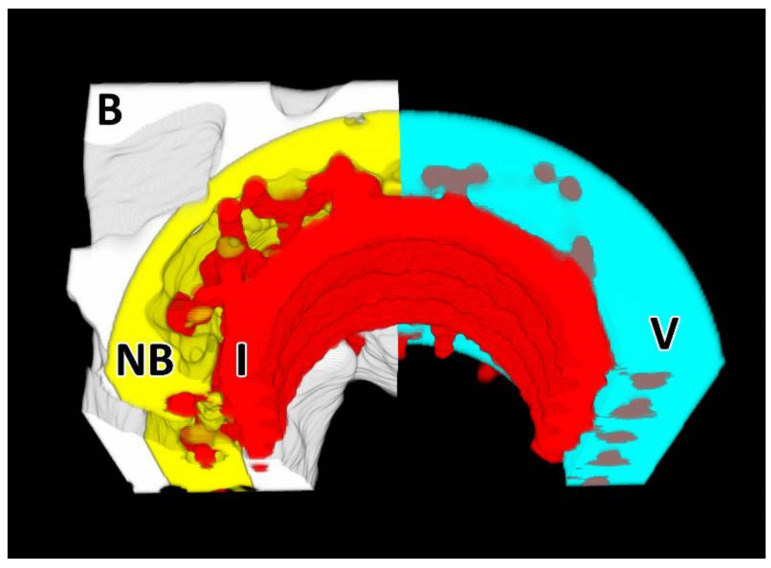
Definition of the micro-CT regions. The VOI (V) consisted of a hollow cylinder with an outer diameter with the same dimensions as the drilled defect and an inner diameter of that of the implant (I) wall. The bone tissue is denoted as B (white) and the “new bone” inside the VOI as NB (yellow).

The micro-CT scanning and image reconstruction procedures were conducted via the same procedure as that applied to the rat femurs. The 3D analysis of the implants was performed by means of CTAn (Bruker, Kontich, Belgium) with the aim of determining the ratio of new bone (RNB) surrounding and penetrating the external surface of the implants. The VOI was set as a hollow cylinder with a border defined by the implant base (the original margins of the drill hole) and the surface of the inner diameter (see Figure 2).

The inner space of the samples (aperture for the inner thread) was excluded from the analysis. Image processing (i.e., noise reduction), binarization, and bit-wise operations (i.e., the subtraction of the implant structure from the bone inside the VOI) were performed prior to the 3D analysis. The ratio of new bone volume was calculated as (2):

(2) $$RNB\;\left(\%\right)=\frac{\mathrm{new}\;\mathrm{bone}\;\mathrm{volume}\;\left(\mathrm{NB}\right)}{\mathrm{VOI}\;\mathrm{volume}\;\left(V\right)\;-\;\mathrm{Implant}\;\mathrm{volume}\;\mathrm{in}\;\mathrm{VOI}\;\left(I\right)}$$

### 2.12. Statistical Analysis

The statistical analysis was performed using statistical software (STATGRAPHICS Centurion XVII, StatPoint, Warrenton, VA, USA). The data is presented in the form of box-and-whiskers plots. A box was drawn extending from the lower quartile to the upper quartile of the sample (this interval covers the middle 50% of the values sorted from smallest to largest). A vertical line was drawn at the median and a plus sign was drawn at the sample mean. Whiskers were drawn from the edges of the box to the largest and smallest data values unless values were situated unusually far from the box. Point symbols outside the whiskers indicate values that were >1.5 times the interquartile range (box width) above or below the box. All the points >3 times the interquartile range above or below the box were termed far outside points and are indicated by point symbols with plus signs superimposed above them. In the case of the presence of outside points, the whiskers were drawn to the largest and smallest data values, which do not constitute outside points. The normality of the data was verified primarily by means of the Shapiro–Wilk’s and Chi-squared tests; outliers were identified via either the Grubbs’ or Dixon’s tests. Homoscedasticity was verified by means of the Levene’s and Bartlett’s tests. The Games–Howell test was applied for the multiple comparison of normally distributed data. Non-parametric analysis was employed since either the assumption of normality or homoscedasticity were violated and, consequently, the Kruskal–Wallis test was applied for a multiple comparison with the subsequent post-hoc test based on the Bonferroni procedure. The Mann–Whitney W test was performed in the case of two-sample comparisons. Statistical significance was accepted at *p* ≤ 0.05.

## 3. Results

### 3.1. The Prevention of the Bone Infection Accompanied by Bone Structure Destruction in the Rat Model

#### 3.1.1. The Infection Potential of *S. epidermidis* Impregnated Implants

The ability of *S. epidermidis* to form a biofilm was proven via a modified Christensen method. The strain was susceptible to oxacillin (MIC = 1 mg/L), linezolid (MIC = 0.5 mg/L), chloramphenicol (MIC = 4 mg/L), tetracycline (MIC = 0.5 mg/L), ciprofloxacin (MIC = 0.5 mg/L), trimethoprim/sulfamethoxazole (MIC = 0.5 mg/L), gentamicin (MIC = 0.5 mg/L), vancomycin (MIC = 1 mg/L) and nitrofurantoin (MIC = 8 mg/L) and resistant to penicillin (MIC = 0.5 mg/L), erythromycin (MIC > 8 mg/L) and clindamycin (MIC = 1 mg/L). All the freshly *S. epidermidis*-impregnated implants (*n* = 5) exhibited 104–106 CFU/mL. However, only 3 of the 16 rat femurs implanted for 7 days with these freshly *S. epidermidis*-impregnated implants exhibited 103 CFU/mL following extraction from the animals, thus indicating the capability of the rat immune system to overcome bacterial contamination and, further, indicating the limitations of this rat model, as discussed in Section 4.

#### 3.1.2. Bone Histology

**Figure 3:**
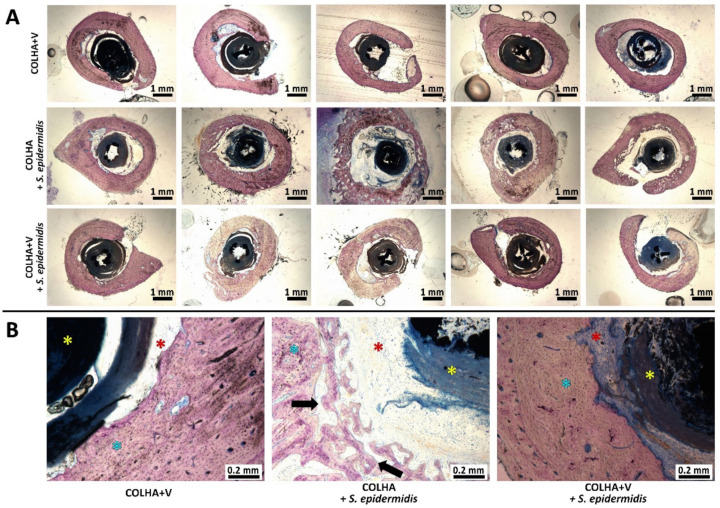
Representative histological images of perpendicular sections of the femurs with Giemsa’s azur eosin methylene blue staining (**A**) and details of the bone morphology (**B**) showing the bone tissue (blue stars), fibrous tissue (red stars), implants (yellow stars), and porous areas (black arrows).

The morphology of the bone surrounding the implants 6 weeks following implantation is illustrated in Figure 3. No signs of the infiltration of bacteria were detected in any of the tested groups six weeks following the introduction of *S. epidermidis* contamination and implantation. The consequences of resolved bone inflammation such as the porosity of the cortical bone were most obvious with respect to the COLHA+*S. epidermidis* group (the black arrows in Figure 3B), whereas the COLHA+V and COLHA+V+*S. epidermidis* groups exhibited only mild or no cortical bone porosity. The fibrous tissue detected in all the groups indicated resolved bone healing (the red stars in Figure 3B). The histological qualitative findings were subsequently quantified via micro-CT and EDS analysis.

#### 3.1.3. Micro-CT Analysis

**Figure 4:**
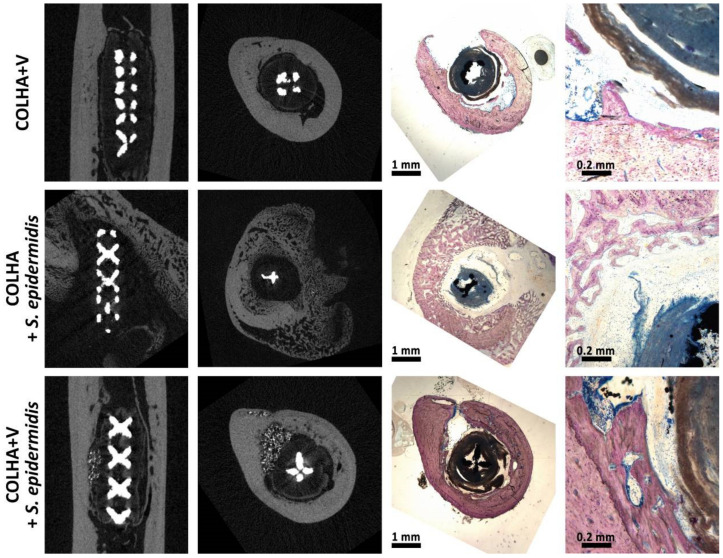
Representative micro-CT and histological images of identical samples in the COLHA+V implants without the application of *S. epidermidis* (upper line), COLHA implants with *S. epidermidis* (middle line), and COLHA+V with *S. epidermidis* (bottom line) groups.

**Figure 5:**
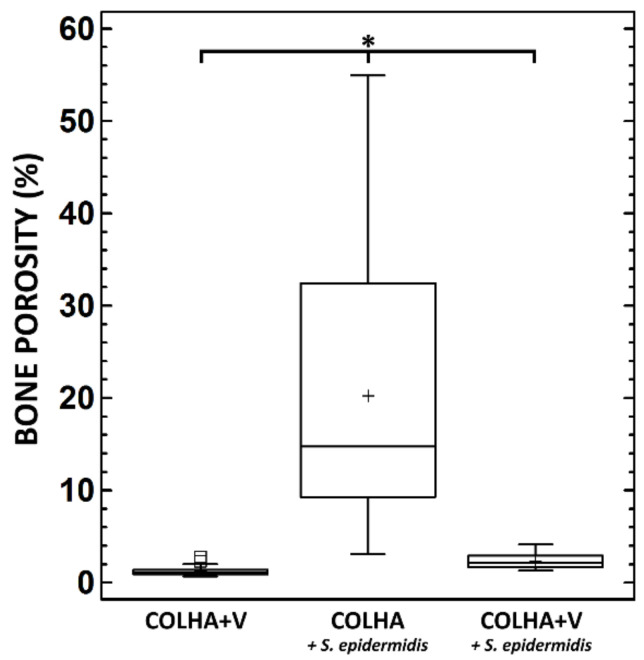
Bone porosity of the rat femurs for the implants with a COLHA+V layer and for the implants with COLHA and COLHA+V layers with *S. epidermidis*. * Denotes statistically significant differences (0.05, Kruskal–Wallis with the Bonferroni procedure).

The evaluation of COLHA+V layers to prevent bone infection accompanied by bone structure destruction was based on the hypothesis that inflammation at the site of implantation would lead to the alteration of the bone structure; hence, cortical bone porosity was selected as the corresponding parameter. The micro-CT visualizations (Figure 4) revealed increases in cortical porosity in the following ascending order: (1) COLHA+V, (2) COLHA+V+*S. epidermidis*, and (3) COLHA+*S. epidermidis*. However, an evaluation of the 2D sections revealed the intraindividual variability of the findings (heterogenous porosity localization and the variable presence of the bone cement material) especially with respect to the COLHA+V+*S. epidermidis* experimental group. Thus, it appears that relatively small changes in the position and orientation of the section may lead to the determination of a different structure. Unfortunately, this represents a limit to conventional 2D approaches (e.g., histology). The cortical bone of the specimens was then subjected to a 3D porosity analysis (Figure 5) that revealed the following mean porosity values: COLHA+V = 1.66%, COLHA+V+*S. epidermidis* = 2.35%, and COLHA+*S. epidermidis* = 19.84%. The highest data variability was observed with concern to the COLHA+V+*S. epidermidis* experimental group. It is important to consider the limitations of this approach relating to the limited spatial resolution of micro-CT, i.e., the possible presence of non-detected and thus non-analyzed pores. However, no other option is yet available for the non-destructive whole-specimen 3D analysis of samples of such a size that provides for a considerably higher resolution than the method used in our study.

#### 3.1.4. SEM and Elemental Analysis

**Figure 6:**
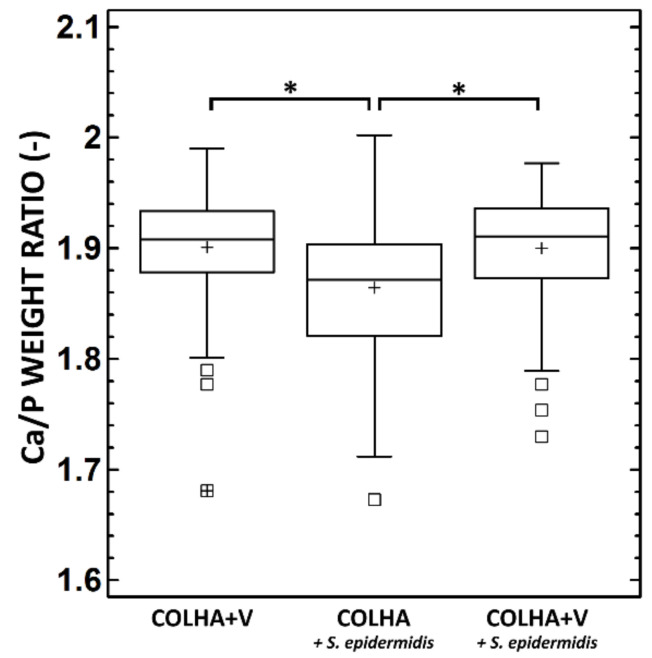
Ca/P weight ratio in the bone tissue of the explanted rat femurs. * Denotes statistically significant differences (0.05, Games–Howell).

Figure 6 provides a summary of the Ca/P weight ratio in the bone tissue of the explanted rat femurs. The average Ca/P ratio of bone mineral in the bone tissue of the explanted femurs with COLHA+V implants without the *S. epidermidis* was 1.90, i.e., the same as that of the COLHA+V implants with *S. epidermidis* (1.89), corresponding to molar ratios of 1.46 and 1.45 respectively. The determined molar ratios were in agreement with those in bioapatites, which were considered to be Ca-deficient if they displayed Ca/P molar ratios of less than 1.67, which is typical for stoichiometric HA. However, the Ca/P molar ratios determined in this study were less than those defined by Bigi et al. (1.5–1.63) [51] and by Termine et al. (1.63) [52] in rat bioapatites. A statistically significant decrease in the Ca/P weight ratio was determined with respect to the COLHA implants with *S. epidermidis* (see Figure 6), i.e., in the case of untreated inflammation (1.85) corresponding to a Ca/P molar ratio of 1.42. The impregnation of the COLHA layer with vancomycin increased the Ca/P molar ratio to the level of that determined in the case of the COLHA+V implants without *S. epidermidis*.

#### 3.1.5. Vancomycin Concentration in the Plasma

**Figure 7:**
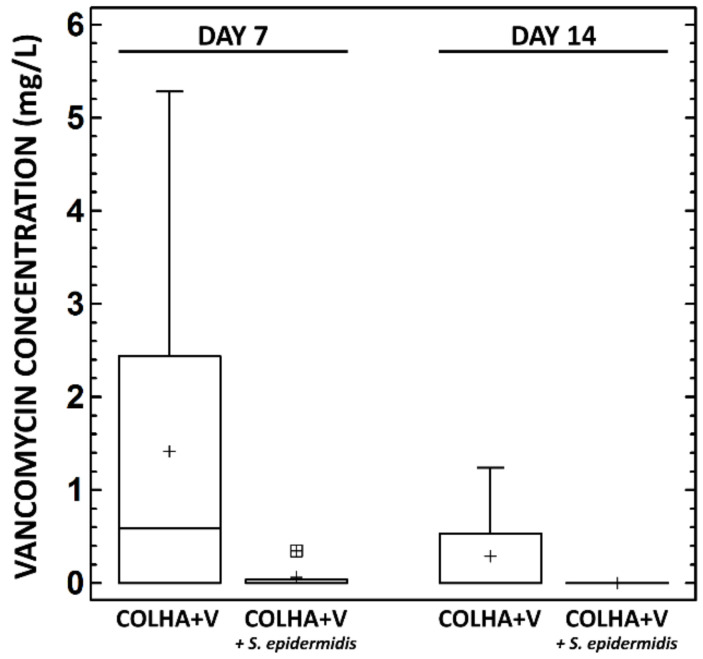
Concentration of vancomycin in the rat blood plasma 7 and 14 days following implantation. Blood samples were taken from the animals with COLHA+V implants with and without *S. epidermidis*. No statistically significant differences were determined between the medians at the 95% confidence level (0.05, Kruskal–Wallis test).

The concentration of the active form of vancomycin released from the implants into the blood was determined 7 and 14 days following implantation (Figure 7).

At day 7, the plasma of the animals without the *S. epidermidis* infection contained 1.42 ± 0.87 mg/L and with the *S. epidermidis* infection 0.07 ± 0.10 mg/L of vancomycin. At day 14, the plasma of the animals without the *S. epidermidis* infection contained 0.29 ± 0.25 mg/L of vancomycin, while no concentration of vancomycin was detected in the plasma of the animals with the *S. epidermidis* infection (Figure 7). While a decrease in the vancomycin concentration in the plasma over time and between the groups with and without infection was observed, the decrease was not statistically significant.

### 3.2. Evaluation of Osseointegration in the Minipig Model

#### 3.2.1. Bone Histology

**Figure 8:**
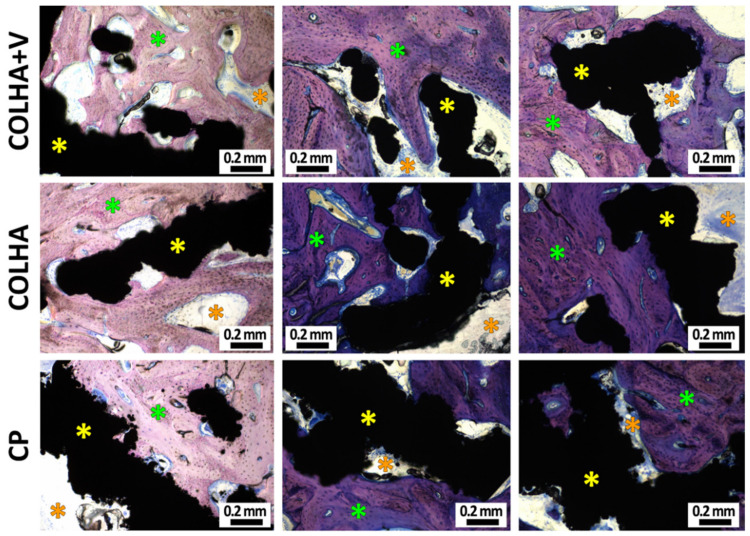
Representative histological images (Giemsa’s azur eosin methylene blue staining) of the printed titanium implants with collagen/hydroxyapatite layers without (COLHA) and loaded with vancomycin (COLHA+V) and the printed titanium control samples (CP). The green stars represent bone tissue outside of the implants, the orange stars represent connective tissue, and the yellow stars the implants.

New bone formation was observed with respect to all three groups of implants six months following implantation (Figure 8). While new bone often formed within the trabecular structure of the implants, new bone formation was found to be more prevalent in the COLHA. Despite the fact that the inner surface of the implants (the hollow part of the thread) was found to be partially surrounded by connective tissue, both the base parts of the hollow cylindrical implants were also observed to be filled with bone tissue. In the case of the COLHA and COLHA+V samples, the porous structure of the implants allowed for bone tissue growth through the lower half of the implant. The qualitative histological results were further quantified via the micro-CT analysis of new bone formation, while the inner part was excluded from the study.

#### 3.2.2. Micro-CT Analysis

**Figure 9:**
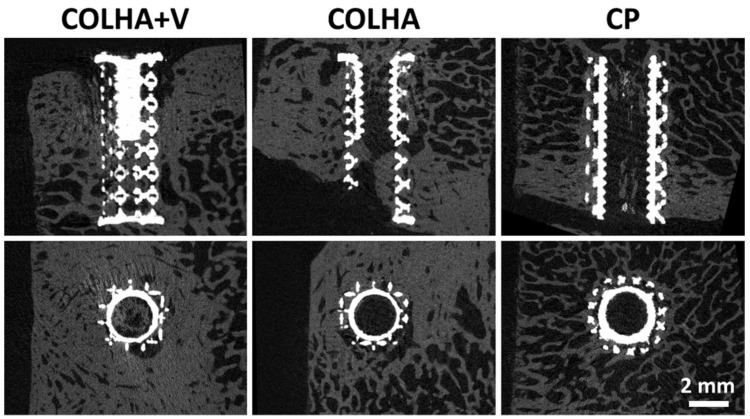
Representative micro-CT images of the implanted titanium samples with collagen/hydroxyapatite electrospun layers with (COLHA+V) and without (COLHA) vancomycin and the control samples without an electrospun layer (CP).

The ratio of new bone (RNB) surrounding and penetrating the external surface of the implant was used in the quantitative comparison of the rate of osseointegration of the pure 3D printed samples (CP) and the 3D printed samples with a COLHA layer and COLHA layer with vancomycin (COLHA+V). Figure 9 presents representative micro-CT images of the three types of titanium implants embedded partly in the cortical bone and partly in the trabecular bone.

**Figure 10:**
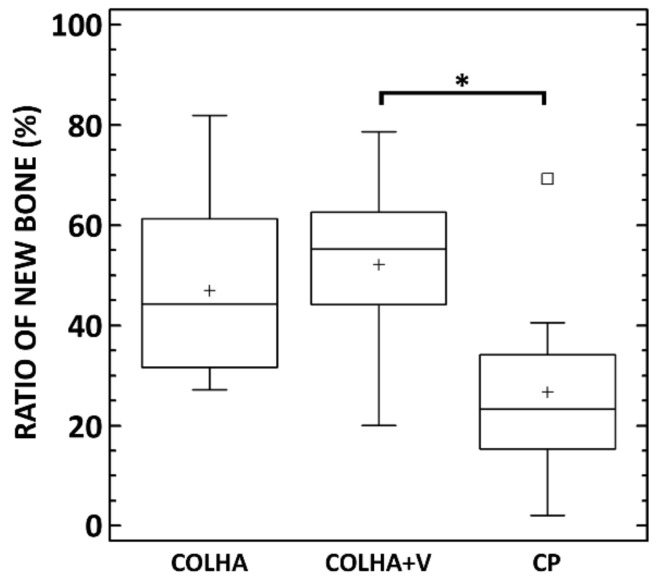
Ratio of new bone integrated into the surface of the implanted titanium samples with collagen/hydroxyapatite electrospun layers with (COLHA+V) and without (COLHA) vancomycin and the control samples without an electrospun layer (CP). * Denotes statistically significant differences (0.05, Kruskal–Wallis test with the Bonferroni procedure).

The micro-CT analysis confirmed that newly-formed bone had grown into the porous structure of all the implants. Moreover, the rate of ingrowth varied depending on the region of the cortical or trabecular bone subjected to assessment. The bone growth results are summarized in Figure 10. The average ratio of new bone increased in the order from the CP to the COLHA and to the COLHA+V samples. While statistically significant differences were observed between the CP and COLHA+V groups, no significant differences were determined between the COLHA and CP and COLHA and COLHA+V groups. The ratio of new bone indicated considerable bone ingrowth (mean, SD) on the COLHA (46.9% ± 17.4%) and COLHA+V (52.2% ± 16.9%) groups compared to the CP control group (26.7% ± 18.4%).

## 4. Discussion

The osseointegration of inert metal implants and their appropriate fixation with bone may be enhanced via the modification of the surface of the implant. Several strategies are applied and aimed at ensuring physical, chemical, and mechanical cohesion between implants and bone. At the same time, such bioactive layers serve as local carriers of antibiotics, the gradual release of which serves for the prevention of osteomyelitis. Ceramic materials, synthetic and natural polymers, and their combinations provide for clinically applied or experimentally developed biodegradable local carriers of antibiotics [18,19,20,21,22,23,24,25,26,27]. The local application of antibiotics is generally preferred since it enables the introduction of higher dosages; moreover, the short transport path and minimal levels of antibiotic fluctuation in the blood stream also represent significant advantages. Thus, antibiotics administered in this way also act in the avascular zones without increasing systemic toxicity. On the other hand, one of the drawbacks of local carriers of antibiotics concerns the inhibition of osseointegration [53]. Thus, both antimicrobial activity and osseoconductivity must be combined in the one implant. To follow this requirement, our team firstly performed the successful assessment of collagen/hydroxyapatite electrospun layers with vancomycin in terms of antibiotic release kinetics, antimicrobial efficiency, and cytocompatibility [3,27]. As the further step, the aim of this study was to conduct a comprehensive in vivo biological evaluation of both the effect of such layers on experimental infection in a rat femur implant-related infection model involving inoculation with a clinically relevant *Staphylococcus epidermidis* strain and the effect of such layers on the osseointegration of implants using a pig model.

The most common microorganisms responsible for implant-associated infection consist of *S. aureus* and *S. epidermidis* [54]. In order to replicate the relevant clinical situation, our study involved the injection of a bacterial inoculum of *S. epidermidis* isolated from a rejected implant into the femoral medullary cavity of rats fitted with 3D printed titanium implants coated with collagen/hydroxyapatite layers with and without vancomycin. Six weeks following experimental infection and implantation, no significant signs of chronic infection were found in any of the experimental groups. However, the rats in the COLHA group with *S. epidermidis* displayed the obvious destruction of cortical bone. An alteration in the bone structure by means of increased bone porosity of up to 20% was evident in the group infected with *S. epidermidis* and treated with COLHA without vancomycin (COLHA+*S. epidermidis*). In contrast, the rats in the infected group treated with COLHA with vancomycin (COLHA+V+*S. epidermidis*) exhibited a bone porosity occurrence of just 3% and those in the uninfected COLHA group of 2%. An increase in bone porosity constitutes one of the markers of bone inflammation and contributes significantly to poor implant osseointegration [55].

It is well known that the various tissues of the human body differ greatly in terms of the proportions of chemical elements. Thus, it can be expected that normal bone, inflamed bone, and bone tumors, being of different tissues have specific and differing elemental compositions as, indeed, was proved by Zaichick and Zaichick [56]. According to the literature, this pathological process is associated with the alteration of the Ca/P ratio and a deficiency of microelements [57]. Several mechanisms of bone loss through osteomyelitis have been mentioned in the literature [58]. Even though bacterial biofilms are known to form via osteomyelitis, direct bacterial attack on bone is believed to make up a negligible mechanism [59]. Esmonde-White et al. [60] have hypothesized that bacterial biofilms are responsible for generating an acidic environment and, subsequently, that if the localized microenvironment cannot be adequately buffered, this environment leads to the dissolution of substituted carbonates and calcium ions. Consequently, a change may occur in the final Ca/P ratio in bone influenced by osteomyelitis. The value of the Ca/P ratio will probably depend (owing to the varying degree of substitution) on the part of the bone analyzed. In this study, a statistically significant decrease in the Ca/P ratio was determined with respect to the COLHA implants with *S. epidermidis*, i.e., in the case of untreated inflammation. This decrease is in accordance with the conclusions formed by Esmonde-White et al. [60] who studied compositional changes in bone infected by the osteomyelitis of the diabetic foot and who proved the presence of pathological minerals such as brushite and uncarbonated apatite. They stated that the acidic environment caused by the osteomyelitis enabled the dissolution of carbonates in the bioapatite and the formation of brushite with a Ca/P molar ratio of 1. The findings of this study proved that the COLHA+V layers are sufficient to prevent bone structure destruction as the consequence of bone infection in vivo.

In order to verify only local vancomycin release from implants, the concentration of the active form of vancomycin in the blood stream was determined 7 and 14 days following implantation. At day 7, the plasma of the animals without *S. epidermidis* infection contained up to 1.4 mg/L and those with *S. epidermidis* up to 0.1 mg/L of vancomycin. At day 14 the plasma of the animals without the *S. epidermidis* infection contained up to 0.3 mg/L of vancomycin, while no concentration of vancomycin was detected in the plasma of the animals with the *S. epidermidis* infection. In the case of the application of vancomycin in the form of continuous infusion, less than 10 mg/L of vancomycin in blood plasma is recommended in order to avoid renal failure or ototoxicity [31]. Our results indicate that vancomycin applied via COLHA layers is capable of remaining locally within the bone with the minimal systemic loading of the organism [61].

Both a high degree of osseointegration and the prevention of infection are required for successful implantation [62]. In order to investigate the effect of collagen/hydroxyapatite layers and vancomycin on bone growth in a pig model, the control pure titanium printed implants were compared to the COLHA and COLHA+V implants. New bone formation was observed with concern to all three groups of implants six months following implantation. The micro-CT analysis quantitatively confirmed that newly-formed bone had grown into the porous structure of all the implants. The ratio of new bone indicated considerable bone ingrowth on the COLHA (47%) and COLHA+V (52%) compared to the CP control group (27%). Alghamdi et al. [63] implanted nano-CaP (synthetic nano-sized crystalline carbonate apatite particles) and collagen-coated (rat tail collagen type I) commercial pure titanium implants in the mandibles of beagle dogs. Three months following implantation, the nano-CaP implants exhibited a bone volume in the inner zone of the implants as assessed by micro-CT of 45.9% ± 9.9% and the collagen-coated implants of 55.9% ± 9.7%. Zhang et al. [64] studied the osseointegration of titanium implants surrounded by biomimetic nanosized hydroxyapatite and collagen prepared via intrafibrillar (IMC) [65] and extrafibrillar (EMC) [66] mineralization. Three months following implantation in the distal femoral condyle of male Sprague–Dawley rats, the micro-CT evaluation of the osseointegration revealed a ratio of the bone volume to the total volume of 41.9% ± 7.7% for the IMC and 19.9% ± 6.1% for the EMC. Jang et al. [67] studied osseointegration titanium implants coated with collagen type I (porcine skin) and collagen mixed with hydroxyapatite and bone morphogenic protein (BMP-2). Based on the histo-morphometric analysis of the samples, which had been implanted for 3 months (New Zealand white rabbits tibia), they demonstrated the significantly greater formation of new bone following the application of the collagen coating (57% ± 3.5%) compared to the uncoated implants (22% ± 6.2%). The incorporation of hydroxyapatite and BMP-2 further enhanced new bone formation up to 87% ± 5.2%. Lee et al. [68] utilized an aerosol deposition system [69] for the coating of titanium implants, which were further coated with collagen type I (calf skin). Six weeks following implantation in the tibia of New Zealand white rabbits, the formation of new bone increased to 47.04% ± 17.82% in contrast to the untreated titanium implants (23.34% ± 13.28%). With respect to our study, the ratio of new bone also indicated the considerable bone ingrowth of the titanium implants following the application of collagen with hydroxyapatite at comparable levels. Moreover, the rate of new bone formation did not decrease following the application of vancomycin. The results of the in vivo biological evaluation suggest that the application of collagen/hydroxyapatite layers improved the rate of osseointegration without the loading of vancomycin acting to impair in vivo bone growth into the implants.

It is important to note our observation that the persistent experimental infection of the rat femurs by *S. epidermidis* applied in this study was detected in only 19% of the animals; the following text discusses the possible causes of this phenomenon. Aimed at precisely simulating the operation procedure and the introduction of bacterial contamination to the bone via implantation, we impregnated the tested implants via the inoculum of *S. epidermidis* and introduced them to the medulla of the rat femurs using an approach applied in other studies [54,70,71,72,73]. *S. epidermidis* was selected since, together with *S. aureus*, it represents the most common microorganism responsible for implant-associated infections [54,73,74]. However, the most probable reason for the low frequency of persistent bone infection detected in this study comprised the animal model itself. Rat species originate from bacterially rich environments and their immune systems are accustomed to combating systematic infections [75]. Further, the selection of *S. epidermidis* instead of S*. aureus*, although they represent two of the most common bacteria responsible for implant-associated infections, may have influenced the low incidence of bone infections observed in our model. Finally, the dose of the implanted colony forming units (CFU) of *S. epidermidis* per bone defect may have played a role, as observed in another study that demonstrated that the healing of fractures in experimentally infected bone defects depended on the bacterial load introduced [76].

## 5. Conclusions

The study demonstrated that collagen/hydroxyapatite layers directly electrospun on the surface of 3D printed titanium implants and impregnated with 10 wt % of vancomycin have the potential to prevent bone destruction as the consequence of bone infection while maintaining osseointegration. The antimicrobial activity of the COLHA+V layers was found to be sufficient to maintain the cortical bone porosity and Ca/P ratios at physiological levels following the experimental infection of the bone using a rat femur implant-related infection model with the inoculation of a clinically relevant *Staphylococcus epidermidis* strain. The in vivo tests employing a pig model concluded that a COLHA+V coating is capable of effectively improving the rate of osseointegration. Both the antimicrobial and osteoinductive functions of electrospun COLHA+V layers help to reduce the revision rate and enhance the long-term success of bone implants. In addition, even though we repeated the implant-related bone infection surgical procedure using *S. epidermidis*, bone infection was not detected in all the animals, thus indicating a limitation to the clinical relevance of the model, which should be considered in further studies.

Future intentions with respect to this study comprise addressing a number of technological issues related to the deposition of the electrospun layers, i.e., their homogeneity, including their dimensional homogeneity, and the homogeneous distribution of the various components such as antibiotics and hydroxyapatite. A further important factor concerns the adhesion of the electrospun layer to the metallic alloy surface. The various steps involved in the use of surface treatments for enhancing adhesion and layer homogeneity were presented in a paper previously published by the authors [39]. Although we are confident that the highly promising results of our study have the potential for application in the field of modern orthopedics, it will be necessary to conduct additional preclinical studies going forward employing large animal sample sizes aimed at verifying our findings.

## 6. Patents

The research reported in this manuscript resulted in the following patent application “A nanocomposite layer based on collagen nanofibers, and a method for the preparation thereof” that was granted in August 2020 by the European Patent Office under the registration number EP3311854.
